# Ageing, Age-Related Cardiovascular Risk and the Beneficial Role of Natural Components Intake

**DOI:** 10.3390/ijms23010183

**Published:** 2021-12-24

**Authors:** Jacek Rysz, Beata Franczyk, Magdalena Rysz-Górzyńska, Anna Gluba-Brzózka

**Affiliations:** 1 Department of Nephrology, Hypertension and Family Medicine, Medical University of Lodz, 113 Żeromskiego Street, 90-549 Lodz, Poland; jacek.rysz@umed.lodz.pl (J.R.); bfranczyk-skora@wp.pl (B.F.); 2 Department of Ophthalmology and Visual Rehabilitation, Medical University of Lodz, 113 Żeromskiego Street, 90-549 Lodz, Poland; mrs-89@o2.pl

**Keywords:** ageing, cardiovascular risk, resveratrol

## Abstract

Ageing, in a natural way, leads to the gradual worsening of the functional capacity of all systems and, eventually, to death. This process is strongly associated with higher metabolic and oxidative stress, low-grade inflammation, accumulation of DNA mutations and increased levels of related damage. Detrimental changes that accumulate in body cells and tissues with time raise the vulnerability to environmental challenges and enhance the risk of major chronic diseases and mortality. There are several theses concerning the mechanisms of ageing: genetic, free radical telomerase, mitochondrial decline, metabolic damage, cellular senescence, neuroendocrine theory, Hay-flick limit and membrane theories, cellular death as well as the accumulation of toxic and non-toxic garbage. Moreover, ageing is associated with structural changes within the myocardium, cardiac conduction system, the endocardium as well as the vasculature. With time, the cardiac structures lose elasticity, and fibrotic changes occur in the heart valves. Ageing is also associated with a higher risk of atherosclerosis. The results of studies suggest that some natural compounds may slow down this process and protect against age-related diseases. Animal studies imply that some of them may prolong the lifespan; however, this trend is not so obvious in humans.

## 1. Introduction

**Figure 1:**
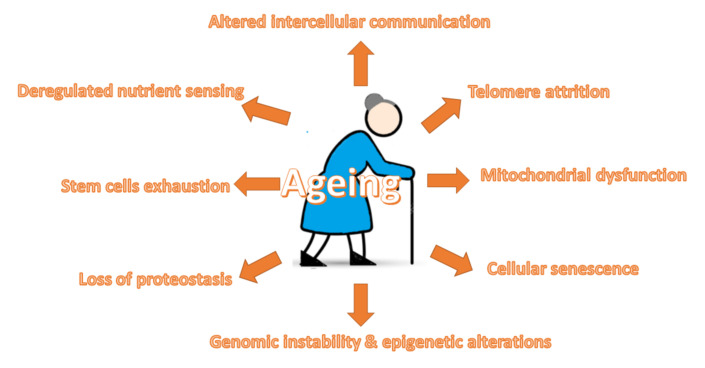
Mechanisms involved in ageing.

Ageing is a natural process that exerts an impact on organism physiology, leading to reduced ability to survive stress as well as progressive functional impairment and death [1,2]. It is a complex and virtually universal biologic phenomenon [3]. In its course, various detrimental changes progressively accumulate in cells and tissues, resulting in the weakening of their functioning, systematic dysfunction of almost all organs, increasing the vulnerability to environmental challenges and enhancing the risk of major chronic diseases, such as cardiovascular disorders, cancer, diabetes, neurodegenerative diseases and mortality [1,4,5,6]. The prevalence of these chronic diseases has been estimated to be responsible for more than 70% of the deaths among Americans 65 years of age and older [4]. Ageing and chronic diseases have been found to be strongly associated with higher metabolic and oxidative stress, low-grade inflammation as well as the accumulation of DNA mutations and increased levels of related damage [7,8]. There are several theories concerning the mechanisms of ageing, including genetic, free radical telomerase, mitochondrial decline, neuroendocrine theory, Hay-flick limit and membrane theories. Cellular senescence, metabolic damage (such as mitochondrial somatic DNA damage, respiratory chain dysfunction and glycation), cellular death as well as the accumulation of toxic and non-toxic garbage (e.g., advanced glycation end products, atherosclerotic and amyloidal plaque, lipofuscin and cortisol) have been suggested to be responsible for the ageing process [1,9,10,11,12]. The presence of mitochondrial dysfunction is associated with the formation of free radicals accelerating the ageing process. In turn, the cellular senescence involves stable or “irreversible” cell cycle arrest, which is induced by, among others, telomere dysfunction, DNA damage, oxidative stress and some types of cytokines [13,14,15]. The body’s antioxidant levels gradually decrease with advancing age, and the signs of ageing start to appear when free radical damage accelerates [1]. Disturbances of the vascular system, hormones as well as cellular and intracellular damage associated with the body’s failure to keep up with the damage result in the mobilization of the body’s repair and immune system to heal the injury or fight off an invader [1]. The process of ageing is stimulated by telomerase shortening, inadequate DNA repair and anti-oxidant system, defective cell cycle control, autophagy and defective proteasome, lysosomes and shock proteins [16,17,18,19]. Ageing is associated with the increased risk of cardiovascular diseases (CVD), which are the prominent causes of death today [20]. According to estimations, more than half of the CVD-related deaths occur in individuals aged 65–74 years [21]. Changes in the cardiovascular system associated with ageing involve decreased heart contractility, impaired diastolic function, calcifications, cholesterol-rich plaque formation, endothelial dysfunction and defective vessels relaxation [22,23]. Age-related low-grade inflammation, oxidative stress, diminished bioavailability of nitric oxide (NO), mitochondrial dysfunction, impaired bioenergetic efficiency, enhanced apoptosis, age-related decline of autophagy, cellular senescence as well as the activation of the renin–angiotensin–aldosterone system appear to be the crucial mechanisms underlying cardiovascular ageing [24,25]. The advancing age is an important risk factor for CVD; however, the results of studies have indicated that a healthy lifestyle and diet have the potential not only to decrease CVD risk but also to prolong the lifespan. Currently, the interest in ageing concentrates on understanding its origins, mechanisms and processes, as well as on the research regarding how to decrease, postpone or reverse its effects [26]. The promotion of healthy ageing, involving the reduction in the risk of the development of major chronic diseases, maintenance of physical and cognitive functions as well as intact mental health and good quality of life, has recently become an important public health issue [6]. Some natural compounds have been suggested to exert beneficial effects on ageing and cardiovascular disease. This review will focus on the mechanisms involved in ageing and ageing-related cardiovascular risk as well as some natural compounds with favourable properties. The summary of the mechanisms involved in ageing is presented in Figure 1.

## 2. Ageing-Related Mechanisms

The results of studies have demonstrated that ageing promotes the development of atherosclerosis even in the absence of other risk factors [27]. Ageing per se was found to be associated with endothelial dysfunction [28]. So far, nine cellular hallmarks of ageing have been identified.

### 2.1. Telomere Attrition

According to studies, telomere attrition is considered as one of nine hallmarks of ageing [5]. It is believed to determine the cellular lifespan [26]. Telomeres are repetitive protective DNA–protein complexes that are located at the ends of eukaryotic chromosomes [6]. These sequences enable the recognition of chromosome ends (and their discrimination from double-strand breaks), thereby protecting chromosomes from recombination, end-to-end fusion and degradation. They also protect the physical integrity of genomic DNA due to the fact that they prevent its loss at the ends of linear chromosomes [29,30]. However, each somatic cell division is associated with telomeres attrition and, therefore, the shortening of telomeres with age is related to diminished life expectancy and enhanced rates of developing age-related chronic diseases [31,32,33]. When telomeres become critically short, replicative senescence is triggered as a result of the activation of a DNA damage checkpoint [34]. Apart from age, oxidative stress and inflammation have an impact on telomere attrition (an accelerating effect) [35,36]. The shortening of telomeres is counteracted by the cellular enzyme telomerase. The shortening of telomeres was found to decrease the regenerative potential of stem cells [34]. Deficiencies in the DNA repair and telomerase pathway components have been found to cause premature ageing in humans and mice [37]. Greater DNA damage and reduced telomere length exert an impact on the development of atherosclerosis, coronary artery disease and heart failure since such phenomena appear to stimulate cellular senescence in the vascular endothelium, thus enhancing the inflammatory cycle and leading to plaque deposition [38,39]. In ageing cardiomyocytes, the attrition of telomeres and DNA damage were demonstrated to be responsible for cell loss associated with higher cellular senescence and apoptosis. In consequence, this process may limit the proliferative potential of cardiac progenitor cells, leading to heart failure [40,41]. According to studies, lifestyle factors that promote the development and progression of cancer and cardiovascular disease may also unfavourably affect telomerase function [42]. Some studies suggest that dietary patterns may be partially responsible for the variability in the telomeres attrition rate; i.e., plant-based foods containing high amounts of compounds exerting antioxidant and anti-inflammatory effects may counteract this process [6]. Polyphenols have been suggested to prevent the shortening of telomeres [43]. Moreover, subjects consuming a Mediterranean diet (MedDiet) rich in olive oil had significantly improved leukocyte telomere length [44]. Proanthocyanidins and procyanidins, due to their potent free radical scavenging and anti-inflammatory properties, were demonstrated to reduce apoptosis and limit the hydrogen peroxide-induced damage of human lymphoblastic cells [45]. In turn, epigallocatechin-3-gallate (EGCG) and quercetin may hamper cardiac myocyte apoptosis by preventing telomere shortening and telomeric repeat-binding factor 2 (TRF2) loss [46]. Quercetin can interact with human telomerase and stabilize the G-quadruplex structure [46]. Due to the fact that accelerated telomere attrition may be the cause of many chronic diseases, the preventive strategies relying on diet would be of particular relevance. Recent studies confirm the relationship between proper diet and telomeres attrition.

### 2.2. Genomic Instability and Epigenetic Alterations

Exogenous chemical, physical and biological agents, but also spontaneous hydrolytic reactions and DNA replication errors, constantly pose a threat to DNA integrity and stability [5,47]. Apart from this, the accumulation of free oxygen radicals, such as reactive oxygen species (ROS) and hydrogen peroxide (H_2_O_2_), could also result in enhanced oxidative damages (carbonylation, glycation, oxidized methionine, DNA damage and the aggregation of proteins) contributing to ageing and age-related diseases [48,49]. Extrinsic or intrinsic damage results in telomere shortening, point mutations, chromosomal gains and losses, translocations and gene disruption caused by the integration of viruses or transposons. However, in our body, there are complex networks of DNA repair mechanisms that are responsible for the maintenance of undamaged DNA [50]. Moreover, mechanisms for preserving the proper length and functionality of telomeres and mitochondrial DNA integrity are necessary for genomic stability [51]. The accumulation of epigenetic defects or epimutations and changes in chromatin architecture throughout life constitute characteristic features of ageing [52,53]. Apart from the aforementioned, the occurrence of premature ageing syndromes may be associated with defects in the nuclear architecture (laminopathies), leading to genome instability [54]. Finally, throughout life, our DNA is subjected to epigenetic alterations involving the post-translational modification of histones, DNA methylation as well as chromatin remodelling [55]. It has been demonstrated that the modified methylation or acetylation of some histones (H4K20 or H3K4 or H3K27 trimethylation, reduced H3K9 methylation or H4K16 acetylation) pose age-associated epigenetic marks [5,56]. According to studies, in invertebrates, the methylation of histones fulfils the criteria for a hallmark of ageing. Histone demethylases have been demonstrated to be able to modulate the lifespan via acting on the components of key longevity routes, including the insulin/insulin-like growth factor (IGF-1) signalling pathway [57]. However, it appears that the association between ageing and DNA methylation is much more complex since there is no direct evidence proving that the altering modification of the DNA methylation pattern can prolong the lifespan [5]. In general, the aforementioned alterations (epigenetic modifications and genomic instability) result in the worsening of the cellular function manifested by organelle dysfunction, disturbed intercellular communication and cellular senescence and lead to chronic inflammation, or “inflamm-ageing”, disturbances in neuronal function, body composition and energy metabolism [5,26,58]. Some natural components have been demonstrated to exert antioxidative effects. Natural potent antioxidants from plant sources involve: polyphenols (phenolic acids, anthocyanins, flavonoids, lignans and stilbenes), carotenoids (carotenes and xanthophylls) as well as vitamins (vitamin E and C) [59,60]. Their beneficial impact has been confirmed in many studies. For example, flavonoids can bind with DNA to form a duplex, which protects DNA from oxidative damage [61]. Moreover, curcumin and jasadbhasma have been shown to prevent DNA damage from free radicals [62]. Moreover, jasadbhasma hampered the production of hydrogen peroxide (43%) by the metal-catalysed glycation system.

### 2.3. Mitochondrial Dysfunction

In the course of the ageing process of cells and organisms, the efficiency of the respiratory chain becomes reduced, which results in greater electron leakage and diminished adenosine triphosphate (ATP) generation [17]. Deteriorated mitochondria were found to accumulate mitochondrial DNA (mtDNA) damage and produce excessive amounts of reactive oxygen species [63,64]. The results of numerous studies confirm the role of mitochondrial dysfunction in ageing and age-related disease. The mitochondrial free radical theory suggested even that the damage resulting from excessive ROS production by mitochondria was the driving force behind ageing [65]. However, some more recent research papers have challenged this theory, indicating an inverse relationship between mitochondrial ROS production and the lifespan in mammals [66]. Low levels of ROS species play important signalling roles; however, excessive and aberrant ROS generation exerts a plethora of detrimental effects, including ageing [64]. Ageing is associated with the dysregulation of the biological systems due to the aggravating impairment in cellular signalling molecules over time [67]. The exact mechanisms underlying ageing are not fully understood; however, it seems that oxidative stress plays a key role in this process, being, at the same time, responsible for the deleterious consequences of human ageing [3]. Oxidative stress weakens the biological structures and repair mechanisms, leading to the imbalance between the rate of cellular damage and renewal of homeostasis and tissue function. Excessive levels of ROS stimulate the oxidation of fatty acids as well as oxidative damage of DNA and subsequent cellular senescence, functional alterations and pathological conditions, including age-related chronic diseases [68,69]. Oxidants react also with proteins, leading to deleterious consequences on protein structure and function. Moreover, protein degrading systems (the proteolytic and lysosomal) have been found to be affected during ageing since their proteolytic activity becomes significantly limited [70]. The formation of reactive carbonyl compounds (RCCs) may promote ‘carbonyl stress’, which, in turn, favours the modifications of cellular proteins and nucleic acids and finally results in mitochondrial dysfunction, apoptosis, the dysfunction of tissues and the progression of diseases, including diabetes, atherosclerosis, etc. [71,72].

According to studies, the mechanisms through which dysfunctional mitochondria contribute to ageing can be independent of ROS [73,74]. It was found that mitochondrial deficiencies may influence apoptotic signalling through the triggering of inflammatory reactions and/or enhancing the propensity of mitochondria to permeabilize in response to stress [17,75]. The adverse effect of flawed mitochondria may also be associated with Ca^2+^ deregulation and altered fusion/fission dynamics [64]. The age-related decreased efficiency in mitochondrial bioenergetics may be a consequence of telomere attrition [76]. The sirtuins may act as a protective system as they are involved in the removal of damaged mitochondria metabolic, the control of mitochondrial function and the rate of ROS production, thus defending against age-associated diseases [5]. Defective bioenergetics may also stem from the accumulation of mutations and deletions in mtDNA, oxidative damage of mitochondrial proteins leading to the destabilization of the organization of respiratory chain (super)complexes, alterations of lipid composition of mitochondrial membranes and compromised quality control by mitophagy [77]. Defective mitochondria are important for the ageing of cardiomyocytes in which decreased energy production translates into impaired contractility [40]. Moreover, the high accumulation of dysfunctional mitochondria elicits apoptosis, which eventually leads to the development of heart failure [78]. Moreover, the excessive production of ROS by mitochondria can promote cellular pathways contributing to pathological myocardial remodelling and subsequent heart failure [79]. The results of animal studies confirm the important role of mitochondrial dysfunction in ageing and cardiovascular system functioning, showing the presence of premature ageing features as well as enlarged hearts in mice with a genetically introduced increased mitochondrial DNA mutation rate [80].

The results of studies have demonstrated that resveratrol, curcumin, oleuropein and hydroxytyrosol can stimulate the up-regulation of mitophagy, thus enhancing both the degradation of damaged mitochondria and the synthesis of new ones [43]. Resveratrol was found to increase the expression of peroxisome proliferator-activated receptor-γ coactivator-1α (PGC-1α) and mitochondrial transcription factor A (mtTFA), which resulted in the improvement in mitochondrial biogenesis and boosted the expression of proteins regulating the balance of mitochondrial fission/fusion and, ultimately, in the maintenance of mitochondrial homeostasis [81,82]. Anthocyanins have been shown to reduce mitochondrial stress [83]. Moreover, delphinidin was found to attenuate the mitochondrial damage associated with the presence of oxidized low-density lipoprotein (oxLDL) [84]. Pyrroloquinoline, acting as a free radical scavenger, preserves mitochondrial function and limits oxidative injury in rat cardiac myocytes [85].

### 2.4. Cellular Senescence

Another hallmark of ageing is cellular senescence, defined as the condition in which the ability of cells to proliferate is arrested and cells undergo profound phenotypic changes [5,58,63]. Senescence can be triggered by telomere shortening but also other ageing-associated stimuli, including non-telomeric DNA damage and de-repression of the INK4/ARF locus [86]. The results of studies have indicated that the state of cellular senescence does not occur in all tissues in aged organisms. It has been suggested that senescence could be a favourable compensatory response enabling the removal of damaged and potentially oncogenic cells from tissues [5]. However, in aged organisms, this system may become ineffective or may deplete the regenerative capacity of progenitor cells, leading in consequence to the build-up of senescent cells, subsequent enhancement of damage and progression of the ageing process. The cells displaying “senescence-associated secretory phenotype” (SASP) are the source of extracellular matrix (ECM)-degrading enzymes [87]. Numerous studies have confirmed increased expression of many metalloproteinases (MMPs), such as MMP-9 and -14 in vascular smooth muscle cells (VSMCs), MMP-1, -3, -8, -10, -11, -12 and -13 in fibroblasts as well as reduced levels of their tissue inhibitors (TIMPs), which accelerate ECM catabolism [88,89,90,91]. Apart from the aforementioned, other ECM-degrading enzymes—a disintegrin and metalloproteinase with thrombospondin motifs (ADAMTSs)—were also found to be up-regulated in senescent cells [87,92]. Senescent cells are also characterized by elevated levels of ECM regulatory enzymes, including cathepsin B (in endothelial cells) and urokinase-type plasminogen activator (uPA) and tissue plasminogen activator (tPA) in ECs, fibroblasts and VSMCs [93,94]. The disturbed ECM maintenance is also associated with higher levels of plasminogen activator inhibitors (PAI) -1 and -2 in ECs. Moreover, some research has indicated that the presence of ‘senescence-associated secretory phenotype’ enriched in pro-inflammatory cytokines (interleukin (IL)-6, IL-8), chemokines, immune modulators, granulocyte-macrophage colony-stimulating factor, fibroblast growth factors, signalling molecules, such as damage-associated molecular patterns and many others, may promote ageing [58,63,95,96]. The results mentioned above support the catabolic phenotype of senescent cells, which is further enhanced by the release of pro-inflammatory molecules. Abnormal focal accumulation of senescent cells producing a pro-inflammatory environment (inflamm-ageing) was found to stimulate the onset of pathological conditions, including endothelial dysfunction [97]. Studies provided the evidence of this phenomenon, demonstrating the presence of abundant senescent cells in diseased vascular walls, e.g., arteries of patients with ischemic heart disease [98,99]. Furthermore, the combination of stress-induced senescence and damage-dependent replicative senescence was found to be important for premature vascular ageing and endothelial dysfunction [99,100]. The presence of the catabolic phenotype related to cellular senescence is in agreement with the thinning of the basement membrane, the loss of cellularity and ECM stiffening observed in aged tissues [101].

Some natural components present senolytic properties. For example, fisetin (flavonoid), possessing anti-inflammatory and senolytic activity, was found to induce apoptosis in senescent cells and without affecting cell proliferation in human umbilical vein endothelial cells (HUVECs) [102]. Both the acute or intermittent therapy of old mice and progeroid with fisetin diminished senescence markers in numerous tissues, accordant with the hit-and-run senolytic mechanism. Fisetin also decreased senescence in human adipose tissue with a cell-type specificity. The treatment of old wild-type mice was demonstrated to restore tissue homeostasis, diminish age-related pathology and prolonged the median and maximum lifespan [102]. Oleuropein aglycone administration ameliorates proteasome activity, thus delaying senescence in human fibroblasts. Moreover, the continuous use of this molecule in the case of early passage human embryonic fibroblasts was found to decrease ROS levels, limit the progression of the senescence phenotype by diminishing changes in morphology characteristic for senescence and reduced cell mortality [103,104,105]. Apart from fisetin and oleuropein aglycone, also quercetin, fisetin, piperlongumine and the curcumin analogue show the capability to selectively kill senescent cells [106].

### 2.5. Loss of Proteostasis

The process of ageing, as well as the development of some ageing-related diseases, is associated with compromised protein homeostasis or proteostasis [5,107]. Numerous quality control mechanisms in the cell are responsible for the maintenance of the stability and functionality of their proteomes. Proteostasis encompasses mechanisms related to the stabilization of correctly folded proteins and the destruction of proteins by the proteasome or the lysosome [108]. Such a system not only prevents the accumulation of damaged components but also assures the constant renewal of intracellular proteins. However, the results of some studies suggested that proteostasis may become weakened with ageing [109]. Apart from the recycling of damaged proteins, as well, organelles are removed in an evolutionarily conserved cellular catabolic process (autophagy) [110]. According to studies, there are three types of autophagy: microautophagy, macroautophagy (autophagy) and chaperon-mediated autophagy (CMA) [111]. It has been revealed that autophagy and/or autophagy-related proteins are involved in the control of mitochondria integrity, ROS generation and NLR Family Pyrin Domain Containing 3 (NLRP3) activation [110]. The age-related deficiency of autophagy was found to stimulate the appearance of the inflammation phenotype in cells. Others indicate marked impairment of stress-induced synthesis of cytosolic and organelle-specific chaperones with ageing [18]. Indeed, the results of animal studies confirmed the influence of chaperone reduction on longevity. Moreover, the process of ageing is associated with the decline in two systems involved in protein quality control: the ubiquitin-proteasome system and the autophagy-lysosomal system [112,113]. In contrast, enhanced autophagy was shown to increase the longevity in calorie-restricted mice, which implies that preserving functional autophagy may delay ageing [4,114,115]. The causes of the age-related decline in the expression of autophagy genes remain unknown [4]. The mechanisms of longevity-increasing autophagy are slightly more studied. It seems that the impact on the lifespan is associated with the removal of toxic proteins and defective mitochondria, inhibition of oncogenic transformation, role in the preservation of stem cells, insulin homeostasis regulation and stimulation of immune function [116]. Autophagy is also important for the cellular maintenance in the myocardium [117]. An age-related decrease was found to be associated with the accumulation of impaired and toxic organelles and proteins, which can lead in consequence to cardiac dysfunction and heart failure [118]. The importance of autophagy was confirmed by experimental studies indicating its stimulation in response to cardiac injury [92]. In contrast, the reduction in autophagy was associated with the development of hypertrophy and heart failure in a mouse model [118]. However, excessive stimulation of autophagy following cardiac damage might also be detrimental, as indicated in a study in which worsened beclin-1 function diminished autophagy induction and also limited maladaptive cardiac remodelling following overload [118]. The results of studies underline the importance of the severe age-associated collapse of proteostasis responses and the resultant accumulation of aggregates that cause cellular toxicity, tissue dysfunction and disease; therefore, it seems that boosting the proteostasis processes with the use of natural compounds may prove to be a promising approach to protect our health [119]. For example, resveratrol and quercetin were found to increase proteasome activity via the stimulation of proteasome subunits expression and proteolysis [120]. Beneficial effects of flavonoids and polyphenols have been suggested to be associated also with antioxidant properties and their impact on the stress response through Nrf-2 signalling, which enhances antioxidant potential and the cellular stress-response, thus preventing cellular damage [121]. Moreover, polyphenols can strengthen the degradation of misfolded and damaged proteins in a direct and indirect manner by boosting the activity and efficiency of the cellular protein degradation machinery [122]. Finally, isoflavones and curcumin can stimulate autophagy [123]. The mechanisms behind autophagy induced by tea polyphenols involve the activation of the mammalian target of the rapamycin pathway (mTOR) [124]. In turn, the administration of EGCC diminishes the activity of negative autophagy regulators controlling apoptosis, thus enhancing autophagy and thus increasing cell viability [125].

### 2.6. Stem Cell Exhaustion

Ageing is also characterized by the diminished regenerative potential of tissues [5]. Such functional attrition of stem cells has been demonstrated in bones, muscle fibres and the mouse forebrain [126,127,128]. The reduction in cell cycle activity of hematopoietic stem cells (HSCs) observed in aged mice correlated with DNA damage accumulation as well as the enhanced expression of cell cycle-inhibitory proteins (i.e., p16INK4a) [129]. Age-related stem cell reduction is also associated with telomere shortening [130]. Not only the diminished proliferation of stem and progenitor cells but also excessive process can be harmful since it hastens the exhaustion of stem cell niches. Indeed, the results of animal studies have demonstrated that the exhaustion of HSCs and neural stem cells resulted in premature ageing [131]. The importance of stem cells in ageing was demonstrated in a study of progeroid mice transplanted with muscle-derived stem cells from young mice. Such a procedure was found to increase the lifespan and ameliorate degenerative changes in these animals even in tissues where donor cells are not found [132]. According to studies, preventing stem cell exhaustion poses a promising strategy for anti-aging therapy [5,133]. Caffeic acid was found to restrain *Drosophila* intestinal stem cell aging via the hampering of oxidative stress-associated c-Jun N-terminal kinase (JNK) signaling. Moreover, oral administration of this compound markedly limited age-associated hyperproliferation in intestinal stem cells [133].

### 2.7. Deregulated Nutrient Sensing

The mammalian somatotrophic axis involves the growth hormone (GH) synthesized by the anterior pituitary, as well as the insulin-like growth factor (IGF-1), the secondary mediator of GH produced in response to it. It has been demonstrated that the IGF-1 axis directly controls the downstream molecules, including phosphoinositide 3-kinase (PI3K), Akt serine/threonine kinase (AKT) and orkhead box transcription factors (FOXO), and it also interacts with the aforementioned ageing-relevant signals, such as sirtuins (SIRT1) and 5’AMP-activated protein kinase (AMPK) [4,134]. The ‘insulin and IGF-1 signalling’ (IIS) pathway is considered as the most conserved ageing-controlling pathway. It targets, among others, the FOXO family of transcription factors and the mTOR complexes (sensing of high amino acid concentrations), which have also been implicated in ageing [134,135]. The results of studies have demonstrated that the occurrence of mutations resulting in the lowering of GH, IGF-1 receptor, insulin receptor or downstream intracellular effectors (aforementioned) is associated with longevity. However, at the same time, the decline in GH and IGF-1 have been reported during normal ageing [37]. The explanation of such contradictory observations is provided by a theory according to which organisms displaying constitutively decreased IIS can live longer due to lower rates of cell growth and metabolism (translating into decreased cellular damage); however, physiologically or pathologically aged organisms reduce IIS in an effort to extend their lifespan [136]. The IIS pathway is also involved in glucose-sensing via AMPK (sensing low energy states on the basis of high AMP levels detection) and sirtuins (sensing low energy states via the identification of high NAD+ levels) [137]. According to studies, adenosine monophosphate activated protein kinase (AMPK) pathway, insulin-like growth factor (IGF) signalling (IIS) pathway, sirtuins and target of rapamycin (TOR) signalling regulate growth, metabolic and ageing processes [138,139,140]. Some recent studies have demonstrated that the activation of AMPKα not only prolongs the lifespan but also delays age-associated functional decline in various species [141,142]. Moreover, the decrease in AMPK activity was associated with age-associated dysfunction of skeletal muscle, blood vessel and the liver [143,144,145]. AMPK emerges as a target for the development of new strategies to prolong the lifespan. In turn, sirtuins stimulate the deacetylation of several downstream molecules, such as Ku70 and p53, which are vital for the initiation of apoptosis [146,147]. Circulating IGF-I levels inversely correlate with mammals’ lifespan; however, the exact mechanisms have not been revealed [148]. It has been demonstrated that intense trophic and anabolic activity, signalled through the IIS or the mTORC1 pathways, are vital accelerators of ageing [5]. The inhibition of TOR activity was found to be favourable during ageing; however, at the same time, it impaired wound healing and was associated with insulin resistance [149]. Sirtuins have been demonstrated to be vital nutrient sensors by which pleiotropic functions may influence the lifespan [150]. According to studies, there is complex crosstalk between dietary components and nutrient-sensing pathways. Some compounds of diet can modulate AMPK, SIRTs and mTOR [151,152]. For example, resveratrol was suggested to be a direct activator of Sirt1 and Sirt5 as well as a weak inhibitor of cytoplasmic sirtuin Sirt2 and mitochondrial sirtuin Sirt3 [153,154,155]. Apart from resveratrol, the flavonoid mulberrin, the xanthone gartanin and the alkaloids quinidine and quinine also act as SIRT1 activators [155]. Moreover, chromenone-derived natural products, including fisetin, orientin, quercetin and vitexin, are vital natural products possessing modulatory effects on different sirtuin isoforms.

### 2.8. Altered Intercellular Communication

The ageing process is also associated with changes at the level of intercellular communication [5]. In the course of the ageing process, we observe the aggravation of the inflammatory state, the weakening of immunosurveillance against pathogens and the occurrence of peri- and extracellular environment changes (worsening mechanical and functional properties of all tissues), all leading to the deregulation of neurohormonal signalling, including renin-angiotensin, adrenergic and insulin-IGF1 signalling. As mentioned above, ageing is associated with the appearance of a phenomenon called ‘inflamm-ageing’ resulting from the accumulation of pro-inflammatory tissue damage, enhanced secretion of pro-inflammatory cytokines by senescent cells, defective autophagy response and increased stimulation of NF-κB transcription factor [156]. A progressing proinflammatory phenotype was found to contribute to a long-term activation of the immune system. Low-grade chronic inflammation plays a vital role in the process of ageing and age-related diseases in older adults [4]. The results of studies indicate increased levels of interleukin 6 (IL-6), C-reactive protein (CRP) and tumour necrosis factor α (TNF-α) in ageing individuals even in the absence of acute infection or other physiological stress [157]. The chronic elevation in proinflammatory molecules was demonstrated to impair the function and integrity of various tissues and organs. According to studies, flawed inflammatory responses play a vital role in atherosclerosis [158]. Natural components have been proven to be excellent scavenger and anti-inflammatory factors that could limit the alterations of intracellular communication. For example, curcumin was found to attenuate exercise-induced oxidative stress and inflammation via the modulation of glutathione (GSH), catalase and superoxide dismutase (SOD) enzymes as well as the inhibition of ROS-generating enzymes, including lipoxygen-ase/cyclooxygenase and xanthine hydrogenase/oxidase [159,160]. Curcumin inhibits the nuclear factor kappa-light-chain-enhancer of activated B cells’ (NF-κB) signalling dependent inflammation; thus, it diminishes the intensity of inflamm-aging. The beneficial effects of curcumin on growth, health and slowing down of aging have been shown in numerous studies [161].

## 3. Impact of Ageing on Cardiovascular System

Ageing is associated with structural changes within the myocardium, cardiac conduction system, the endocardium as well as the vasculature [162]. This process induces significant structural alterations, such as vascular stiffening, fibrosis and increased left ventricular (LV) wall thickness, resulting in diastolic dysfunction, greater afterload and, finally, in the development of heart failure with preserved ejection fraction (HFPEF) [163]. Ageing-induced progressive degeneration of the cardiac structures, involving the loss of elasticity (heart thickening and stiffening), fibrotic changes in the valves of the heart and infiltration with amyloid, significantly affects the heart’s left ventricular wall contractility. Moreover, the accumulation of fat around the sinoatrial node may translate into partial or complete separation of the node from the atrial tissue. Atrioventricular conduction block may develop with increasing calcification on the left side of the cardiac skeleton [162]. The ageing heart is characterized by greater cardiomyocyte size, which increases myocardial thickness [164]. The increase in the average myocyte size translates into higher heart mass with ageing; however, at the same time, the number of cardiomyocyte cells becomes reduced, probably due to apoptosis [165]. Greater cardiomyocyte size accounts for left ventricular hypertrophy (LVH) during ageing [162]. Moreover, the change in the heart shape from elliptical to spheroid due to reduced LV length is frequently observed [166]. At that time, the aorta dilates rightward, extending into the cavity of the left ventricle, which, in consequence, leads to a higher wall stress [165,167]. In women, greater LV wall thickness counterbalances the diminishing LV length; however, in men, LV wall thickness fails to compensate, which leads to reduced LV mass with age. The alterations in the shape and thickness exert an impact on heart functioning, including cardiac wall stress and overall contractile efficiency. Some compensatory responses and functional modifications developing with age result in the heart’s reduced ability to respond to amplified workload and lowering of its reserve capacity [162]. These alterations affect heart contractility, maximal heart rate, end-diastolic volume (EDV), end-systolic volume (ESV), sympathetic signalling as well as prolong systolic contraction and diastolic relaxation. Moreover, the rise in valvular circumference in all four cardiac valves has been reported. Apart from this, calcific deposits are frequently observed [167].

Despite many age-related changes, in healthy ageing individuals, the overall resting systolic function of cardiac muscle as well as net systolic function at rest appear to remain unchanged [162]. Ageing is also associated with decreased early diastolic filling. Such a reduction is, to some extent, compensated for by changes in adrenergic signalling, diminishing the maximal heart rate; however, they are not sufficient to preserve the cardiac functional reserve in the case of the exposition to maximal exercise [162]. Therefore, the effects of ageing become more visible in the form of reduced exercise tolerance, which starts at the age of 20 to 30 and falls by approximately 10% per decade [168]. Ageing is associated with reduced autonomic modulation of the heart rate, LV contractility and arterial afterload resulting from diminished efficiency of post-synaptic β-adrenergic signalling [163].

Furthermore, the mechanisms that are responsible for heart protection against injury as well as those involved in injury repair become dysfunctional with ageing [162]. The impairment of these mechanism leads ultimately to enhanced dysfunction and adverse remodelling.

**Figure 2:**
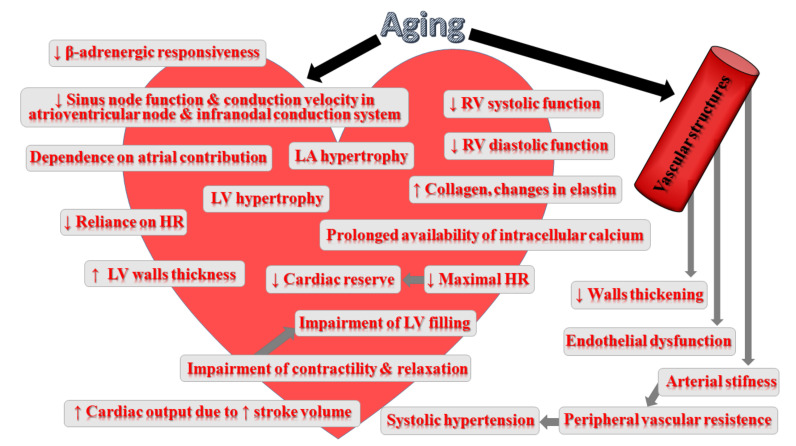
The impact of ageing on cardiovascular system. Abbreviations: HR- heart rate; LA—left atrium; LV—left ventricle; RV—right ventricle, ↑ - increased; ↓ - decreased.

Ageing is associated with reduced elasticity of the arterial vessels, which may lead to chronic or residual rises in vessel diameter and vessel wall rigidity, impairing the function of the vessel. Moreover, collagen accumulation, diminished elastin and enhanced calcification result in greater vascular wall thickening and stiffening [169]. Age-associated changes occur not only in the wall of the aorta but also in the more peripheral vessels. The thickening of walls of veins is due to an increase in connective tissue and calcium deposits. Ageing is connected with functional, structural and mechanical alterations in arteries that strictly resemble the vascular changes underlying the pathogenesis of hypertension [25]. Both structural and functional ageing-associated changes in the heart and vasculature may have severe implications for cardiovascular disease. The presence of low-grade inflammation in the absence of other significant medical conditions (‘inflamm-ageing’) could enhance the susceptibility to CVD in ageing individuals [170]. Increased levels of senescent cells due to ageing could stimulate the secretion of more proinflammatory cytokines. Indeed, the PolSenior study of Eastern Europeans aged ≥65 years demonstrated that the rise in the concentration of IL-6 and CRP occurred in an age-dependent manner [171]. In turn, other studies confirmed the relationship between the higher levels of CRP and both the development of cardiovascular diseases and high risk of CVD mortality [172]. Ageing is also associated with a higher risk of atherosclerosis due to the accumulation of various species of lipids and inflammatory cells within the arterial walls and endothelial injury and dysfunction [173,174]. Chronic excessive production of ROS/reactive nitrogen species (RNS) with ageing can be damaging also for the cardiovascular system [175]. In the heart, the worsening of the mitochondrial respiratory chain function has been identified as one of the key reasons for this phenomenon [176]. Moreover, the impairment of mitochondrial energetics and function appears to be a major determinant in ageing-related cardiovascular disease [177]. Figure 2 summarizes the impact of ageing on the cardiovascular system.

## 4. Impact of Natural Compounds on CVD Risk

Various compounds that scavenge free radicals (such as resveratrol, astaxanthin, gallic acid, etc.) have been suggested to increase longevty or decrease the prevalence of age-related diseases [178,179]. More than 300 compounds with anti-ageing and cardioprotective properties have already been identified [49]. Among the most popular, there are: curcumin, resveratrol, catechin, metformin, α-lipoic acid, fucoxanthin, astaxanthin, rapamycin, spermidine and caffeine [49,180,181,182,183]. Phytochemicals comprise polyphenols, organosulfides, indoles/glucosinolates/sulfur compounds, protein inhibitors, betalians, terpenes and other organic acids [4,184]. These compounds have been found not only to protect and treat chronic diseases (cardiovascular disease, cancer, diabetes, obesity and neurological dysfunctions) but also to exert antiaging effects. Moreover, plant sterols, natural phytochemicals resembling cholesterol, have been shown not only to diminish CVD risk but also to exert anti-inflammatory, antioxidant, anti-atherogenic and anti-cancer properties [1].

### 4.1. Resveratrol

Resveratrol (3,5,4′-trihydroxystilbene) is a small polyphenolic molecule present in various berries, grapes, red and white wine, cocoa, peanuts, pistachios, cranberries, blueberries and dark chocolate [185]. According to studies, it possesses anti-inflammatory, antioxidant and cytoprotective properties [186]. Other studies have indicated that the supplementation of dietary resveratrol exerts favourable effects on chronic diseases, such as cancer, diabetes and Alzheimer’s disease, as well as ageing [187,188]. Resveratrol interacts with various targets in cardio- and cerebrovascular diseases, cancer, age-related diseases, etc. [189,190,191]. The mechanism behind its beneficial biological effects is mediated via the 5′-adenosine monophosphate-activated protein kinase (AMPK)/silent mating type information regulation-1 (SIRT-1) pathway [192,193]. Resveratrol was shown to directly activate SIRT1, which was associated with enhanced energy metabolism mitochondrial biogenesis, insulin sensitivity and survival of high-fat-fed mice [154]. Resveratrol (50 μM) was shown to reverse the oxidative stress-caused decrease in GSH and SOD levels in mice or rats [194]. Frankel et al. [195] observed that the resveratrol contained in red wine inhibited the oxidation of low-density lipoproteins, thus protecting its consumers against coronary heart diseases. It was shown to act at the very early stages of atherosclerosis. Its favourable effects involved the decrease in the expression of intercellular adhesion molecule-1 (ICAM-1) and vascular cell adhesion molecule-1 (VCAM-1) on endothelium as well as enhancing hepatic uptake of low-density lipoprotein (LDL) via an AMPK-independent mechanism [196,197]. In in vitro studies, resveratrol inhibited the expression of MCP-1 and chemokine receptor type 2 in monocytes through the phosphatidylinositol 3′-kinase (PI3K)/protein kinase B (PKB or Akt) pathway [198,199]. Furthermore, Voloshyna et al. [200] revealed that attenuated lipid accumulation and foam cell formation in cultured human macrophages via effects on apoA-1-and HDL-mediated cholesterol efflux and the downregulation of oxidized LDL (ox-LDL) uptake. Moreover, they observed that resveratrol modulated the expression of the cholesterol metabolizing enzyme cytochrome P450 27-hydroxylase, thus enabling effective cholesterol elimination through the formation of oxysterols. Other short-term studies revealed the improvement of insulin resistance, blood flow and the reduction in inflammation and oxidative stress following resveratrol administration [201,202]. Resveratrol-stimulated inhibition of LDL oxidation, macrophage migration and conversion into foam cells, and VSMCs migration and proliferation may be associated with its anti-inflammatory and antioxidant properties [203]. A hypocholesterolemic effect was observed following the administration of a standard dose of resveratrol (20 mg/kg/day) [204]. Resveratrol’s beneficial effects were found to also be mediated via NO and the antioxidant enzyme heme oxygenase-1 (HO-1) [205]. Moreover, it enhances the expression of vascular endothelial growth factor (VEGF) in cardiomyocytes and in endothelial cells via the stimulation of oxidative-stress related proteins thioredoxin-1 (Trx-1) and HO-1 expression [206]. Resveratrol was demonstrated to induce autophagy via different signalling pathways, depending on the cellular and environmental context, thus protecting cardiac tissue from cell death [52,207]. Gurusamy et al. [208] found that pre-treatment of male Sprague Dawley rats with resveratrol (2.5 mg/kg/day gavaged for 2 weeks) resulted in enhanced regeneration of infarcted myocardium (LAD occlusion) reflected by increased cell survival and differentiation. These effects were associated with increased expression of nuclear factor-E2-related factor-2 (Nrf2) and redox effector factor-1 (Ref-1). Moreover, the authors demonstrated the improvement of cardiac functional parameters, including left ventricular ejection fraction and fractional shortening [208]. In turn, Mukhopadhyay et al. [209] reported the restoration of altered microRNAs expression in the ischemic heart following resveratrol administration. Moreover, another study revealed that high doses of resveratrol (50 mg/kg/day) partly reversed left ventricular dilation (reverse remodelling) and markedly improved cardiac function in a mice model of post-infarction heart failure. They also suggested that the effects of resveratrol were dose-dependent. Finally, high doses of resveratrol (320 mg/kg/day) were found to increase animal survival via promoting favourable remodelling and improving diastolic function and cardiac energy metabolism in a mice model of pressure-overload heart failure [210]. However, even low doses (10 mg of resveratrol capsule/day) markedly improved diastolic function and stimulated a modest increase in systolic performance in patients with HF of ischemic origin [211].

### 4.2. Cocoa Extracts

Cocoa extract can be found in cocoa powders, chocolate, brewed cocoa, chocolate spreads, cocoa butter, dark chocolate, chocolate liquor, etc. [212]. Cocoa contains flavanol glycosides, catechins as well as procyanidins and anthocyanins, which exert a pleiotropic influence on various biomedical markers and clinical endpoints of cardiovascular health [213]. Moreover, it is a rich source of polyphenols—approximately 6 to 8% by dry weight—while unprocessed fresh cocoa beans contain about 12–18% [214]. The antioxidant activity of cocoa beans is higher even compared to green tea, red wine and blueberries [212]. Cocoa products are also rich in caffeine, theobromine, theophylline and other methylxanthine compounds, which contributes to their bitter taste [215]. According to studies, active components of cocoa beans exert anti-inflammatory, anti-cancer, anti-hypertensive and anti-diabetes effects, as well as improve the heart condition, relieve stress, enhance cognitive abilities, etc. [212]. The majority of studies of cocoa extracts/products focus on their impact on oxidative stress, plasma antioxidant capacity, nitric oxide metabolism and activity, endothelium-dependent vasomotor function, arterial flow mediated dilatation (FMD), blood pressure, lipid profile, platelet function and vascular inflammation [213,216,217].

Apart from improving cardiovascular function, they also facilitate endogenous repair mechanisms [218]. Cardioprotective properties of cocoa has been suggested to be associated with its antihypertensive, anti-atherogenic and anti-inflammatory properties, as well as the inhibition of the platelet activation and aggregation and improvement of endothelial dysfunction [213]. Cocoa inhibits platelet activation and aggregation. It was found that theobromine contained in cocoa affected platelet aggregation via the inhibition of phosphodiesterase and the increase in cyclic adenosine monophosphate (cAMP) [219]. As a rich source of polyphenols, it exerts strong antiradical and antioxidant properties [20]. Epicatechin contained in cocoa was demonstrated to enhance plasma antioxidant capacity and prevent peroxidation of lipids in the erythrocyte membrane [213]. Cocoa also decreases the production of reactive oxygen species in activated leukocytes [220]. However, some studies in healthy human subjects fail to observe changes in oxidative stress biomarkers following cocoa consumption. The results of trials assessing the effects of cocoa polyphenols intake demonstrated the improvement in the cardiovascular state [221]. The consumption of a flavanol-rich cocoa drink decreased plasma levels of markers of lipid peroxidation, F2-isoprostanes and provided a consequent boost in antioxidant activity [222]. Moreover, polyphenols contained in cocoa were shown to protect LDL particles against oxidation and, therefore, to hamper atherosclerotic progression [223,224]. Cocoa polyphenols improve the lipid profile and promote antiaterogenic effects. A diet containing various concentrations of cocoa powder (0.5–10%) or cocoa extract (600 mg/kg per day) for 4 weeks was associated with the decrease in triglycerides and LDL and (TG) levels, LDL oxidability as well as the increase in the high-density lipoproteins (HDL) and plasma antioxidant capacity in normal rats and hypercholesterolemic rabbits [214]. Cocoa polyphenols affect several inflammatory mediators and signalling pathways in patients with an increased cardiovascular risk [225]. Apart from ameliorating endothelial dysfunction, arterial function and preventing atherosclerosis, anti-inflammatory properties of cocoa polyphenols contribute also to the diminished risk of atherothrombotic clinical syndromes [213]. Another atheroprotective effect involves the stimulation of greater NO bioavailability in the endothelium, which translates into promotion of NO-induced vascular smooth muscle cells relaxation and subsequent vasodilation [226]. The promotion of increased plasma levels of nitric oxide and improvement of flow-mediated vasodilation was confirmed in many human studies assessing the impact of cocoa beverages [227,228]. Repeated administration of cocoa was demonstrated to bring long-term effects characterized by an enhanced baseline level of FMD resulting from the alteration of gene expression and protein synthesis (endothelial nitric oxide synthase, eNOS) [229]. Beneficial endothelial effects of cocoa can also be related to the decrease in xanthine oxidase and myeloperoxidase activities, the modulation of PGI2 and leukotrienes, the inhibition of proinflammatory cytokines IL-1β, IL-2 and IL-8 production as well as ET-1 release [230]. Cocoa may also induce the decrease of monocyte CD62L expression and the increased formation of endothelial microparticles and the mobilization of functionally unaltered circulating angiogenic cells (EPCs) [218,223]. Cocoa polyphenols also have the ability to reverse endothelial dysfunction in CVD [226]. Furthermore, the interaction of cocoa flavonoids with myeloperoxidase results in the inhibition of myeloperoxidase-mediated peroxidation of LDL [231]. Flavanols contained in cocoa were also found to diminish the inflammatory process, thus reducing cardiovascular risk [216]. The Flaviola Health Study showed that flavanols contained in cocoa improved endothelial function and influenced cardiovascular biomarkers, which implies that they may support the maintenance of cardiovascular health even in low-risk subjects [232]. The effect of cocoa related to the reduction in cardiovascular risk is associated with its impact on inflammation, LDL oxidation, lipid peroxidation, lipid and glucose metabolism and BP [233,234]. The mechanisms of the favourable impact of dietary cocoa involve also the improvement in insulin sensitivity [4,235]. Beneficial properties of cocoa have been confirmed in many epidemiological studies. The Iowa Women’s Health Study demonstrated that chocolate consumption reduced mortality from coronary heart disease in postmenopausal women, while the European Prospective Investigation into Cancer and Nutrition found a relation with diminished rate of myocardial infarction and stroke in [236,237]. Cocoa flavonoids participate also in the downregulation of cellular eicosanoid synthesis, which translates into ameliorated vascular tone, inhibition of both platelet aggregation and the recruitment of immune cells into the vascular wall [238,239]. The results of epidemiological studies of the population of San Blas Island who consume great amounts of cocoa beverage indicated decreased prevalence of diabetes, stroke and ischemic heart disease as well as significantly higher lifespan compared with those living in the mainland of Panama [240,241]. What is interesting is that the relocation from San Blas Island to Panama City (and consequent decrease in the consumption of cocoa) was associated with the disappearance of beneficial effects of epicatechin on human health and lifespan [240,241]. Apart from cocoa, tea is a rich and certainly more commonly used source of epicatechin worldwide than cocoa. According to the European Food Safety Authority (EFSA), 200 mg of cocoa polyphenols as a part of the diet is recommended to be consumed daily (contained in 2.5 g of polyphenol-rich cocoa powder or 10 g of polyphenol-rich dark chocolate) in order to improve endothelium-dependent vasodilation [242].

### 4.3. Quercetin

Quercetin (3,3,4,5,7-pentahydroxyflavone) belongs to a group of flavonoids. It is present in large amounts in fruits and vegetables, such as citrus fruits, blueberries, grapes, cherries, blackberries, apples, broccoli onions, parsley, red wine, sage, tea, olive oil, grapes, dark cherries, blueberries and bilberries [4,243]. Chondrogianni et al. [244] found that quercetin and its derivative—quercetin caprylate—act as a proteasome activator with anti-oxidant properties and could affect cellular lifespan, survival and viability of HFL-1 primary human fibroblasts. Moreover, they demonstrated rejuvenating effects after the supplementation to already senescent fibroblasts as well as the stimulation of physiological alterations when applied to cells. Therefore, it seems that these natural compounds can be used as effective anti-ageing products following their topical application [244]. Apart from the scavenging of reactive oxygen species, quercetin has been revealed to possess strong anti-inflammatory capacity as it is capable of hindering lipopolysaccharides-induced production of interleukin-1α and tumour necrosis factor-α in immune cells [245,246]. Both antioxidant and anti-inflammatory properties may be associated with the observed antiaging effect of quercetin and its derivatives [4]. Beneficial effects of quercetin have been observed in *Saccharomyces cerevisiae* and in *Caenorhabditis elegans* in which it boosted resistance to oxidative stress and prolonged the lifespan [247,248]. Apart from its impact on lifespan, quercetin was also found to be beneficial for the prevention and treatment of CVD due to antioxidant, anti-inflammatory, vasodilatory and anticlotting properties [249,250,251]. The modulation of mitochondrial functionality appears to be one of the vital protective mechanisms [252]. The results of both animal and human studies confirmed the ability of this flavonoid to inhibit the platelet aggregation and prevent the endothelial dysfunction, thus hampering the development of CVD [253,254,255]. Quercetin-induced endothelial protection involves the increase in nitric oxide bioavailability, the regulation of p47phox expression (and subsequent modulation of NOX activation) as well as the inhibition of superoxide production [254]. The maintenance of endothelial function translates into vasorelaxation and blood pressure lowering, even in already hypertensive humans [256]. Numerous studies have provided evidence of beneficial effects of quercetin on atherosclerosis development by interfering with various pathways involved in disease progression [257]. The results of studies of animal models fed with a high-fat diet indicated that, following the exposure to quercetin, animals showed reduced atherosclerotic plaque areas [258,259]. Quercetin-promoted protection against atherosclerosis was suggested to be associated with its impact on the expressions of ATP-binding cassette transporter (ABCA1), liver X receptor α (LXR-α) and proprotein convertase subtilisin/kexin 9 (PCSK9) in ApoE-/- mice [218]. In turn, Jia et al. [259] suggested that the effects of quercetin were related to the regulation of the expression of PCSK9, CD36, peroxisome proliferator-activated receptor (PPARγ), liver X receptor α (LXRα) and ABCA1 in apoE-/- mice. Experiments on rodent models and murine cultured macrophages demonstrated that quercetin stimulated cholesterol-to-bile acid conversion and cholesterol efflux by upregulating the activity of hepatic CYP7A1, liver X receptor α, ABCG1, ABCA1 and LDLR [257,260]. Mice that received quercetin during exercise sessions were found to have 78% atherosclerotic plaque reduction compared to control mice and 40% less atherosclerotic plaque formation compared to a control group supplemented with quercetin [261]. Moreover, the down-regulation of the expression of MMP-1, MMP-2 and MMP-9 by quercetin was found to prevent against plaque instability [262,263]. Moreover, the anti-aggregatory effects of this compound seem to protect against acute complications of atherosclerosis resulting from the aggregation of platelets at the site of an unstable plaque [264]. The development of atherosclerosis is also associated with the presence of a. chronic inflammatory state characterized by increased levels of IL-1α, IL-1β, IL-2, IL-10, TNF-α, macrophage chemoattractant protein-1 and cyclooxygenase-2 [265]. Additionally, in this aspect, the administration of quercetin was shown to be beneficial. Its effects involved the reduction in inflammatory cytokines. A double-blind clinical trial in which patients were administered quercetin at the dose of 150 mg/day for 6 weeks demonstrated the decrease in systolic blood pressure and plasma oxidized LDL levels in overweight subjects at a high-cardiovascular risk [249]. The effect of quercetin on total and LDL cholesterol was not associated with changing triglyceride levels [266]. In turn, the use of quercetin in an in vitro model of posttraumatic cardiac dysfunction was associated with the decrease in cardiomyocyte apoptosis via the inhibition of TNF-α increases, ROS overproduction and Ca^2+^ overload in cardiomyocytes [267]. Histopathological examinations revealed the protection of cardiomyocytes’ membrane integrity and global enhancement in myocardial function after exposure with quercetin. These beneficial effects were associated with decreased infiltration of leukocytes to the site of infarction, maintained overall tissue architecture as well as reduced oedema [268,269]. The reduction in Src kinase activity, caspase 9, signal transducer and activator of transcription 3 (STAT3), intracellular ROS production as well as inducible MnSOD expression are other mechanisms related to cardioprotective effects of quercetin [270]. Moreover, Liu et al. [271] observed significant deceleration in LVEF decline and fractional shortening in quercetin-fed mice compared with the control group.

### 4.4. Curcumin

Regarding curcumin (diferuloylmethane), this lipophilic phenolic compound belongs to curcuminoids that are present in curcuma plants: turmeric (*Curcuma longa*) [272]. Turmeric extracts can be included in “functional” food and beverages, such as bread, biscuits, cheese, snacks, milk, fresh sausage, pasta and patties [273]. Curcumin is comprised of three forms: curcumin I (94%), curcumin II (6%) and curcumin III (0.3%) [274]. For many centuries, curcumin has been used in traditional Chinese and Indian medicine to treat some disorders, such as jaundice, dysentery, wounds ulcers, arthritis, acne as well as skin and eye infections [275]. Kitani et al. [276] observed that the addition of tetrahydrocurcumin (a metabolite of curcumin) to mice food significantly improved their survival [276]. Curcumin exerts antioxidant and anti-inflammatory properties [277,278]. As a powerful antioxidant, curcumin neutralizes free radicals, thus limiting their damaging effects to every cell and especially the cell’s DNA [1]. The addition of curcumin (0.5–1.0 mg/g) to the culture medium extended the life span of Drosophila flies by 10%. This effect was associated with the reduction in oxidative stress, lipid peroxidation and decreased accumulation of dialdehydes as a result of the modulation of various stress-responsive genes [279]. According to studies, the dietary intake of curcumin (2 mg/g medium) enhanced SOD activity by 32% in *Drosophila*, while the administration of curcumin (8 mg/kg, intraperitoneal injection for 5 days) to mice was able to restore X-ray-reduced hepatic SOD and GSH contents [280,281]. Curcumin not only upregulates the sirtuin pathway but also activates the Nrf2-ARE pathway [282]. There is increasing evidence of a potential role of curcumin in protection from CVDs [283,284]. The effects of curcumin are mediated by various molecular targets, including ERK, MAPK p38, Janus Kinase 2 (JAK2)/STAT3, AMPK/UCP2, Akt/Nrf2, JNK, MCP-1, ICAM-1 and IL-8 [189,285,286]. Therefore, it possesses anti-inflammatory, antiplatelet and antioxidant properties [287,288]. Even a single dose of curcumin (15 mg/kg) was found to reduce superoxide anion, xanthine oxidase, myeloperoxidase, lipid peroxides as well as to enhance the concentration of glutathione-S-transferase (GST), catalase, SOD and GpX [289]. Apart from this, curcumin protects the endothelium via the induction of heme oxygenase-1 (HO-1) and iNOS through the activation of NF-κB and protein-1 (AP-1) [290,291]. Moreover, the exposition to curcumin was found to have antiproliferative and antiapoptotic effects on VSMCs [292]. Curcumin seems also to diminish mitochondrial alterations and respiratory cellular dysfunction [292]. The results of animal studies confirm the hypolipidemic effect and protection from aortic fatty streak development and thus the cardioprotective properties of curcumin [293,294]. Moreover, this natural compound diminished the synthesis of collagen, limited fibrosis and markedly enhanced ejection fraction, left ventricular end-diastolic volume and stroke volume in Sprague Dawley rats subjected to ischaemia followed by reperfusion and, therefore, inhibited maladaptive cardiac repair and maintained cardiac function [295]. Other studies reported that the administration of curcumin prevented myocardial hypertrophy via the inhibition of p300-HAT (histone acetyltransferases) as well as protected against adriamycin-induced cardiac damage [296,297].

### 4.5. Carotenoids

Carotenoids (xanthophylls and carotenes) are naturally occurring compounds produced by plants, fungi, several bacteria and plastids of algae [298,299]. Nearly 600 carotenoids have been identified in nature, but only 50 of them can be found in the human diet, and about 20 are present in human tissues and blood [298,300]. Due to the fact that these compounds can absorb wavelengths between 400 and 550 nm, their colour is usually red, orange or yellow [301]. Orange–yellow vegetables and fruits are rich sources of β-carotene and α-carotene, while orange fruits, tomatoes and tomato products and dark green vegetables contain α-cryptoxanthin, lycopene and lutein, respectively [302]. Egg yolk is a source of zeaxanthin and lutein [303].

Carotenoids have an electron-rich conjugated system of the polyene structure; thus, they can scavenge the free radicals via trapping peroxyl radicals and quenching the singlet oxygen [304]. Favorable effects of carotenes are also associated with their antioxidant activity [305]. The results of studies revealed that a high intake of carotenoids was inversely associated with the occurrence of age-related diseases [306]. Their consumption was demonstrated to prevent oxidative stress-induced diseases, including CVD [307]. The Coronary Artery Risk Development in Young Adults (CARDIA)/Young Adult Longitudinal Trends in Antioxidants (YALTA) study revealed the beneficial impact of carotenoids on inflammation (leukocyte count, C-reactive protein), markers of oxidative stress (circulating extracellular superoxide dismutase) and endothelial dysfunction (soluble P-selectin, soluble intercellular adhesion molecule-1 (sICAM1)) [308]. In animal studies, all-trans β-carotene was found to be able to inhibit atherosclerosis in a manner that was independent of LDL-C resistance to oxidation [309]. Plasma concentrations of α- and β-carotene appeared to inversely correlate with the risk of carotid and femoral artery atherosclerosis [310]. The results of a Finnish study carried out on Kuopio Ischaemic Heart Disease Risk Factor (KIHD) cohort indicated that low serum β-carotene concentrations were associated with increased CVD mortality risk. In this study, men in the lowest quartile of β-carotene levels showed a 2-fold higher risk of CVD mortality compared with those in the highest quartile [311,312].

Lutein, possessing strong anti-inflammatory and antioxidant properties, diminishes the risk of cardiovascular disease coronary artery disease and CVD in elderly populations [313,314]. The consumption of one soft boiled egg (lutein) every day for 4 weeks was found to decrease the levels of oxidized low-density lipoprotein and thus to hamper the development of atherosclerosis [315]. In turn, the consumption of three eggs for the period of 30 days was associated with no significant difference in the total amount of LDL or HDL particles, but it increased the less atherogenic LDL lipoprotein subfractions and enhanced high-density lipoprotein (HDL) functionality in hyper-responders [316]. Moreover, it has been suggested that high plasma concentrations of lutein can prevent ischaemic injury of myocardium via the reduction of oxidative stress and apoptosis and diminish the risk of stroke and coronary heart disease [313,317]. Moreover, lycopene exerts beneficial vascular, endothelial and cardiac protective effects [318]. Low plasma lycopene was reported in middle-aged men with subclinical atherosclerosis assessed on the basis of the increase of intima-media thickness of the common carotid artery (CCA-IMT) [319]. Moreover, an independent inverse relationship was found between serum levels of lycopene and one of the markers of arterial stiffness (brachial-ankle pulse wave velocity) [319].

Some studies indicated a beneficial impact of high serum concentrations of carotenoids on serum NT-pro BNP levels, implying that they may prevent cardiac overload [320]. Patients with high plasma levels of β-cryptoxanthin and lutein had a lower risk of acute myocardial infarction [321,322]. Furthermore, a study of patients with coronary artery disease revealed decreased concentrations of lutein, zeaxanthin, β-cryptoxanthin, α-carotene, β-carotene and lycopene compared to healthy individuals [323]. The reports of an inverse association between plasma provitamin A carotenoids and matrix metalloproteinase-9 may imply that these nutrients can hamper the degradation of the extracellular matrix in the arterial wall [324]. Table 1 presents the results of studies regarding the impact of natural compound on cardiovascular risk.

## 5. Conclusions

Ageing is a natural process leading to the gradual worsening of the functional capacity of all systems and, ultimately, death. Detrimental changes that accumulate with time in body cells and tissues are associated with the systematic dysfunction of almost all the organs, increasing the risk of major chronic diseases, such as cardiovascular disorders, cancer, diabetes, neurodegenerative diseases and mortality. Despite growing knowledge on CVDs, its prevalence continues to increase; therefore, there is a need for new effective and safe products. Increasing evidence provided by in vitro and in vivo studies has implied that some natural compounds, including resveratrol, curcumin, quercetin and carotenoids, may modulate vital cellular, molecular and metabolic mechanisms involved in the pathogenesis and progression of CVD. Indeed, the results of studies suggest that some natural compound may slow down this process and protect against age-related diseases, including cardiovascular disease. However, it should be kept in mind that many complex and multifactorial processes are involved in both aging and HF and, therefore, it is plausible that agents targeting only a single pathway will not be able to fully mitigate age-related cardiac phenotypes. Some natural components have a wide range of beneficial actions (antioxidant, anti-inflammatory, etc.) and seem to be more safe. However, in some cases, problems with poor bioavailability have been demonstrated in clinical trials. Moreover, the results of studies with the use of natural compounds are sometimes conflicting. This can be associated with the inclusion of different groups of patients but also with the difficulty to accurately establish the content of a given chemical in a given fruit or vegetable. On the other hand, the extraction techniques may deprive the compound of its beneficial properties.

Animal studies have implied that some natural compounds may also prolong the lifespan; however, this trend is not so obvious in humans due to the fact that humans’ life span is too long to observe effects.

## Figures and Tables

**Table 1:** The results of studies regarding the impact of natural compound on cardiovascular risk.

Name of Compound/Dose	Study Type/Study Group	Effects	Ref.
**Resveratrol**	In vitro/cells of the arterial wall, including human macrophages and arterial endothelium.	- Regulates the expression of key proteins involved in cholesterol transport - Stimulates apoA-1 and HDL-mediated efflux, - Downregulates oxLDL uptake and diminishes foam cell formation. - These effects depend on PPAR-γ and adenosine 2A receptor pathways. - Regulates expression of the cholesterol metabolizing enzyme 27-hydroxylase—thus improves the efficacy of cholesterol elimination via formation of oxysterols. - Hampers lipid accumulation in cultured human macrophages via effects on cholesterol transport.	[200]
**Resveratrol**/ resVida™ 30, 90 and 270 mg and a placebo at weekly intervals	Double-blind, randomized crossover comparison 19 overweight/obese men or post-menopausal women with untreated borderline hypertension	- Significant dose effect of resveratrol on FMD (*p* < 0.01) (4.1 ± 0.8% (placebo) and 7.7 ± 1.5% after 270 mg resveratrol). - Acute resveratrol consumption increased FMD in a dose-related manner.	[201]
**Resveratrol** (2 × 5 mg resveratrol p.o.) or placebo.	4-week-long double-blind randomized study/ 19 patients	- Significantly decreased insulin resistance and urinary ortho-tyrosine excretion - Increased the pAkt:Akt ratio in platelets. - Resveratrol improves insulin sensitivity in humans, which might be due to a resveratrol-induced lowering of oxidative stress, which results in more efficient insulin signalling via the Akt pathway.	[202]
**Resveratrol** (0.5 to 1 mg/kg body wt),	Animal study/I/R rat heart	- I/R-induced iNOS induction is abrogated - Markedly upregulated expression of eNOS and nNOS. - Protective effects on I/R rat heart comprise: decreased rhythm disturbances, diminished cardiac infarct size and reduced plasma levels of lactate dehydrogenase (LDH) and creatine kinase (CK). Reductions in LDH/CK levels and infarct size are NO-dependent - Decrease in the severity of ventricular arrhythmia and mortality rate are mediated by NO-independent mechanism.	[205]
**Resveratrol** (2.5 mg/kg/day gavaged for 2 weeks)	Animal study/male Sprague Dawley rats (pre-treated)	- One week after the LAD occlusion, enhanced expression of Nrf2 and Ref-1 was seen in resveratrol-treated rat hearts - Significantly improved cardiac functional parameters (LVEF and fractional shortening). - Improvement of cardiac function was accompanied by the increased stem cell survival, proliferation and differentiation towards the regeneration of the myocardium	[208]
**Resveratrol** (pure compound) or longevinex (commercial resveratrol formulation)	Animal model/ischemia/reperfusion model of rat (pre-treated)	- Unique pattern of miRNA expression in hearts of animals pre-treated with resveratrol (pure compound) and longevinex (commercial resveratrol formulation). - Differential expression of over 25 miRNAs, including miR-21 implicated in cardiac remodelling. - Target genes for the differentially expressed miRNA were associated with metal ion binding, sodium-potassium ion, transcription factors, which may play key role in reducing I/R injury.	[209]
10 mg **Resveratrol** capsule daily for 3 months	Double-blind, placebo randomized controlled trial 40 post-infarction Caucasian patients	- Left ventricular ejection fraction showed a tendency to increase in treated group. - Significant improvement of left ventricular diastolic function (*p* < 0.01). - Considerable improvement in endothelial function (measured by FMD) (*p* < 0.05). - Markedly diminished low-density lipoprotein (LDL) level (*p* < 0.05). - Prevented against decrease in red blood cell deformability and increase in platelet aggregation.	[211]
Dietary high-**Flavanol** intervention (HiFI 375 mg) and a macronutrient- and micronutrient-matched low-flavanol intervention (LoFI 9 mg) twice daily in random order over 30 days.	Randomized, controlled, double-masked, cross-over trial, 16 CAD patients	- Improvement of endothelium-dependent vasomotor function (by 47%) in the HiFI period compared with the LoFI period. - Increase in the amount of CD34+/KDR+-CACs after HiFI compared with after LoFI. - Reduced systolic blood pressure (mean change over LoFI: −4.2 ± 2.7 mm Hg) - Increased plasma nitrite level (mean change over LoFI: 74 ± 32 nM).	
High-**Flavanol** cocoa drink (HFCD; 187 mg flavan-3-ols/100 mL) vs. low-flavanol cocoa drink (LFCD; 14 mg/100 mL).	Comparative randomized double-blind crossover study/20 volunteers were examined in a design with respect to ingestion of	- LFCD caused a slight increase in the mean plasma concentrations of F(2)-isoprostanes, which may be attributable to postprandial oxidative stress. No increase in HFCD group - Dietary flavanols can lower the plasma level of F(2)-isoprostanes, the indicators of in vivo lipid peroxidation.	[222]
Natural **cocoa**-containing product (12.7 g natural cocoa) or an isocaloric cocoa-free placebo daily for 4 weeks with a 2-week washout period between treatment arms.	Randomized, double-blind study/24 young women consumed a	- Significant decrease in haptoglobin (*p* = 0.034), EMP concentration (*p* = 0.017) and monocyte CD62L (*p* = 0.047) in obese compared to overweight and normal-weight subjects. - 18% increase in high-density lipoprotein (*p* = 0.020) and a 60% decrease in EMPs (*p* = 0.047) in general. - Decreased obesity-related disease risk.	[223]
Meals supplemented with 1.4 g of **cocoa extract** (645.3 mg of polyphenols) vs. control meals—15% energy restriction diet.	4 week randomised, parallel and double-blind study/50 healthy male and female middle-aged volunteers	- Higher reduction of oxidised LDL cholesterol (*p* = 0.030) in the cocoa group. - Decreased myeloperoxidase (MPO) levels only in the cocoa supplemented group (*p* = 0.007). - Cocoa intake was more beneficial effects in men.	[224]
Acute, single-dose ingestion of cocoa, containing increasing concentrations of flavanols (75, 371 and 963 mg). 30-day, thrice-daily dietary intervention with either **flavanol-rich cocoa** (321 mg flavanols per dose) or a nutrient-matched control (25 mg flavanols per dose).	Feasibility study/10 diabetic patients Efficacy study/41 medicated diabetic patients	- Single ingestion of cocoa was associated with significant acute increases in circulating flavanols and FMD in a dose-dependent manner (*p* < 0.001). - 30-day consumption of cocoa increased baseline FMD by 30% (*p* < 0.0001) - Treatment was well tolerated without evidence of tachyphylaxia. - Endothelium-independent responses, blood pressure, heart rate and glycemic control remained unaffected.	[227]
**Cocoa flavanol**-containing drink (450 mg) or a nutrient-matched cocoa flavonol-free control bi-daily for 1 month.	Randomised, controlled, double-masked, parallel-group dietary intervention trial/100 healthy, middle-aged men and women	- Increased FMD compared to control - Reduced systolic and diastolic blood pressure by 4.4 mmHg (95% CI 7.9, 0.9 mmHg) and 3.9 mmHg (95% CI 6.7, 0.9 mmHg) and pulse wave velocity - Decreased total cholesterol and LDL-cholesterol, - Increased HDL-cholesterol - Predicted a significant lowering of 10-year risk for CHD, myocardial infarction, CVD, death from CHD and CVD. - Regular intake improved accredited cardiovascular surrogates of cardiovascular risk, which suggests the potential to maintain cardiovascular health even in low-risk subjects.	[232]
150 mg **quercetin**/d during 6-week treatment periods separated by a 5-week washout period vs. placebo	Double-blinded, placebo-controlled cross-over trial/93 overweight or obese subjects aged 25–65 years with metabolic syndrome traits. Subjects were randomised to receive in a	- Decreased systolic blood pressure by 2.6 mmHg (*p* < 0.01) in the entire study group, by 2.9 mmHg (*p* < 0.01) in the subgroup of hypertensive subjects and by 3.7 mmHg (*p* < 0.001) in the subgroup of younger adults aged 25–50 years. - Reduced serum HDL-cholesterol concentrations (*p* < 0.001) - Unaltered total cholesterol, TAG and the LDL:HDL-cholesterol and TAG:HDL-cholesterol ratios - Significantly decreased plasma concentrations of atherogenic oxidised LDL - No impact on affect TNF-alpha and C-reactive protein	[249]
150 mg or 300 mg **quercetin**-4’-O-beta-D-glucoside supplement	Pilot human dietary intervention study	- Inhibition of platelet aggregation after 30 and 120 min from ingestion of both doses of quercetin-4’-O-beta-D-glucoside. - Inhibition of collagen-stimulated tyrosine phosphorylation of total platelet proteins - Reduction in tyrosine phosphorylation of the tyrosine kinase Syk and phospholipase Cgamma2, components of the platelet glycoprotein VI collagen receptor signalling pathway.	[253]
High-fat diet (HFD) or a HFD supplemented with 0.05% *w*/*w* **quercetin** (HFD+Q), for 14 weeks.	Animal study/Wild-type C57BL/6 (WT) and apolipoprotein E gene knockout (ApoE(-/-)) mice	- HFD+Q significantly improved endothelium-dependent relaxation of aortic rings isolated from WT but not ApoE(-/-) mice - Reduction in hypochlorous acid-induced endothelial dysfunction in aortic rings of both WT and ApoE(-/-) mice. - HFD+Q significantly improved plasma F2-isoprostanes, 24 h urinary nitrite and endothelial nitric oxide synthase activity and increased heme oxygenase-1 (HO-1) protein expression in the aortas of both WT and ApoE(-/-) mice (*p* < 0.05). - Protection against oxidant-induced endothelial dysfunction and ApoE(-/-) mice against atherosclerosis. These effects are related to the improvements in nitric oxide bioavailability and are arterial induction of HO-1.	[255]
**Quercetin**	Animal study/diet induced hyperlipidemia and aortic atherosclerosis, and in injured carotid artery in rabbits fed high-fat diet for 12 and 4 weeks, respectively	- Reduced serum triglycerides and cholesterol levels elevated by high-fat diet (after 12 weeks) - These effects were less visible in the 4-week study in injured carotid artery rabbit model. - Decreased formation of atherosclerotic plaques, both in the aorta (12-week study) and within injured carotid artery (4-week study) in high-fat diet-fed animals (surface of the intima covered with atherosclerotic plaques in high-fat diet-fed rabbits was 24.6 ± 33.1% vs. 0.7 ± 1.3% (*p* < 0.05) in quercetin and high-fat diet supplemented animals).	[258]
**Quercetin**	Animal study/animal model of high-fat diet induced atherosclerosis—ApoE-/- mice	- Prevents the development of AS in apoE-/- mice by regulating the expression of PCSK9, CD36, PPARγ, LXRα and ABCA1	[259]
**Quercetin**, GW9962 (PPARγ antagonist) or PPARγ-siRNA alone or in combination (pre-treatment)	Animal study/Myocardial IRI in mice or to hypoxia and reoxygenation (H/R) treatment in H9C2 cells	- Significantly improved cardiac function, diminished myocardial injury and reduced the infarct size (quercetin) - Markedly improved myocardium oxidative damage and apoptosis in vivo and in vitro. - Suppressed activation of the NF-κB pathway induced - GW9662 or PPARγ knockdown partially reduced these cardioprotective effects of quercetin during myocardial IRI.	[271]
**Curcumin**	Animal study/Swiss albino mice receiving metronidazole, 40 mg/kg bw and 13.4 mg/kg bw × 3 days exposure	- Increased the levels of hepatic GSH and SOD	[281]
Single oral dose of **curcumin** (15 mg/kg), administered 30 min before and/or after the onset of ischaemia	Animal study/isoprenaline induced myocardial ischaemia in rat myocardium	- Pre- and post-treatment decreased levels of xanthine oxidase, superoxide anion, lipid peroxides (LPs) and myeloperoxidase - Markedly increased levels of superoxide dismutase (SOD), catalase (CAT), glutathione peroxidase (GPx), glutathione-S-transferase (GST) activities. - Significant improvement of severe myocardial damage due to isoprenaline induced ischaemia - Protection of rat myocardium against ischaemic insult, and the protective effect could be attributed to its antioxidant properties and its inhibitory effects on xanthine dehydrogenase/xanthine oxidase (XD/XO) conversion and consequent superoxide anion production.	[289]
**Curcumin** at the dose of 150 mg/kg/day only during reperfusion	Animal study/Sprague Dawley rats were subjected to 45 min of ischaemia followed by 7, 21 and 42 days of reperfusion	- Reduced the level of malondialdehyde - Inhibition of MMPs activity - Protection of ECM from degradation and attenuated collagen deposition, reduced extent of collagen-rich scar and higher mass of viable myocardium. - Reduced collagen synthesis and fibrosis in the ischaemic/reperfused myocardium - Significant down-regulation of TGFβ1 and phospho-Smad2/3 expression and up-regulation of Smad7 - Considerably improved left ventricular end-diastolic volume, stroke volume and ejection fraction. - Greater wall thickness of the infarcted middle anterior septum compared to control group.	[295]
**Carotenoids**	4580 black and white men and women in the Coronary Artery Risk Development in Young Adults study	- sICAM1 concentrations were higher in the highest carotenoid quartile in smokers than in the lowest carotenoid quartile in non-smokers. - Superoxide dismutase positively correlated with the sum of 4 carotenoids (*p* < 0.01). - Lycopene inversely correlated only with sICAM1.	[308]
1% probucol, 0.01% vitamin E, 0.01% all-trans **beta-carotene** or 0.01% **9-cis beta-carotene**	Animal study/male New Zealand White rabbits fed a high-cholesterol diet or the same diet with supplements	- Probucol protected LDL from oxidation and inhibited lesion formation. - Vitamin E modestly inhibited LDL oxidation but failed to prevent atherosclerosis. - Beta-carotene had no effect on LDL oxidation ex vivo, but all-trans isomer inhibited lesion formation. - Metabolites derived from all-trans beta-carotene limited atherosclerosis development in hypercholesterolemic rabbits, possibly via stereospecific interactions with retinoic acid receptors in the artery wall.	[309]
**Carotenoids**	1031 Eastern Finnish men in the Kuopio Ischaemic Heart Disease Risk Factor (KIHD) cohort	- Low serum concentrations of β-carotene strongly correlated with increased CVD mortality risk after adjustment for confounders. - Strongest risk of CVD mortality was among smokers with lowest levels of β-carotene (HR = 3.15, 95%, CI: 1.19–8.33; *p* = 0.020). - Lack of relation between other carotenoids and increased risk of CVD mortality.	[311]
**Carotenoids**	1031 Eastern Finnish men in the Kuopio Ischaemic Heart Disease Risk Factor (KIHD) cohort	- Men in the lowest tertile of serum concentrations of β-carotene had a 2-fold increased risk of SCD (HR = 2.15, 95% CI: 1.02–4.51; *p* = 0.044) compared to those in the highest tertile. - Low serum β-carotene concentrations increased the risk of cardiovascular disease (CVD) and total mortality.	[312]
**Lycopene**	Healthy women (*n* = 264, 31–75 yrs)	- Individuals in lycopene tertile showed higher LDL particle size (24.3 ± 0.08 nm vs. 24.0 ± 0.07 nm, *p* = 0.005) and lower C-reactive protein (hs-CRP) (0.80 ± 0.25 mg/dL vs. 1.27 ± 0.24 mg/dL, *p* = 0.015) compared with those in T1. - The presence of an independent inverse relationship between circulating lycopene and brachial-ankle pulse wave velocity.	[319]
**Lutein**	Systematic review with meta-analysis of 71 articles and 387,569 participants.	- Lower risk of coronary heart disease (pooled RR: 0.88; 95% CI: 0.80, 0.98) and stroke (pooled RR: 0.82; 95% CI: 0.72, 0.93) in individuals with the highest compared with the lowest tertile of lutein blood concentration or intake. - Higher lutein level was associated with a lower risk of metabolic syndrome (pooled RR: 0.75; 95% CI: 0.60, 0.92) compared with the lowest tertile.	[317]

LVEF—left ventricular ejection fraction; Nrf2—nuclear factor-E2-related factor-2; Ref-1—redox effector factor-1.

## Data Availability

Not applicable.
